# Intestinal Ischemia Secondary to Ladd’s Bands Obstruction Associated With Intestinal Malrotation in a One-Year-Old Pediatric Patient: A Case Report

**DOI:** 10.7759/cureus.111021

**Published:** 2026-06-17

**Authors:** Benjamin Moya-Leal, Martín Rodríguez-Garza, Cynthia L Nava Palomo, Alejandro Arzamendi, Jose L Valdez-Cruz

**Affiliations:** 1 General Surgery, Institute for Social Security and Services for State Workers, Monterrey, MEX; 2 General Surgery, Instituto de Seguridad y Servicios Sociales de los Trabajadores del Estado (ISSSTE) Clínica Hospital Constitución, Monterrey, MEX

**Keywords:** bowel obstruction, case report, intestinal ischemia, intestinal malrotation, ladd's bands, pediatric surgery, urgent laparotomy

## Abstract

Intestinal malrotation is a congenital anomaly resulting from an alteration in the rotation and fixation of the midgut during embryonic development. Ladd's bands are abnormal peritoneal adhesions that can cause extrinsic compression of the bowel, obstruction, and vascular compromise, leading to intestinal ischemia. Although the diagnosis is typically made in the neonatal period, late presentations exist, posing a diagnostic and therapeutic challenge.

We describe the case of a previously healthy one-year-old male patient who was admitted for feeding intolerance, accompanied by vomiting, progressive abdominal distension, and abdominal pain with clinical signs of peritoneal irritation. Laboratory tests showed an elevated C-reactive protein (71 mg/L) level without significant alterations in the acid-base balance or marked lactate elevation. An abdominal CT scan revealed a bowel obstruction with a transition zone in the terminal ileum. Given the clinical deterioration, an emergent exploratory laparotomy was performed, identifying ischemia in approximately 100 cm of the terminal ileum secondary to Ladd's bands.

Ladd's bands constitute an infrequent but severe cause of bowel obstruction in pediatrics. Early diagnosis and prompt surgical intervention are essential to reduce morbidity and mortality and to preserve the greatest possible bowel length, thereby avoiding bowel resections.

## Introduction

Intestinal malrotation is a congenital anomaly resulting from the interruption of the 270° counterclockwise rotation of the midgut around the superior mesenteric artery during embryogenesis. This alteration leads to inadequate mesenteric fixation and an anomalous position of the bowel loops, and it can progress to ischemia or perforation in advanced or misdiagnosed cases [1].

Like in the study, Strouse et al. [2] say that during normal embryological development, the midgut returns to the abdominal cavity at approximately the 10th week of gestation and completes its counterclockwise rotation around the superior mesenteric artery. Subsequent fixation of the mesentery anchors the duodenojejunal junction to the left upper quadrant and the cecum to the right lower quadrant. When this process is disrupted, the cecum frequently remains in an ectopic position, usually within the right upper abdomen, leading to the formation of abnormal peritoneal attachments known as Ladd's bands. These fibrous bands extend from the ectopic cecum to the retroperitoneum, crossing the duodenum and producing varying degrees of extrinsic compression. The resulting narrow mesenteric base not only predisposes to duodenal obstruction but also increases the risk of midgut volvulus through twisting of the mesenteric vessels.

The incidence of malrotation is around 1 in 500 live births, while symptomatic malrotation occurs in 1 in 6000 births instead [3]. Interruption or alteration of the embryological development of the midgut can occur during any of the previously described phases, either due to "non-rotation" (the most common type), incomplete rotation (which carries a higher risk of complications), or reversed rotation [4].

In the study, Sala et al. [5] say that Ladd's bands are fibrous peritoneal bands that extend from the malrotated cecum to the right retroperitoneum, crossing the duodenum and causing extrinsic compression of the bowel. Their primary risk lies in the potential for bowel obstruction, midgut volvulus, and ischemia due to compromised mesenteric blood flow, sharing a clinical behavior similar to that of malrotation.

Although most cases manifest in the neonatal period with bilious emesis, late presentations in infants can be fatal. We present the case of a one-year-old infant with extensive intestinal ischemia secondary to Ladd's bands.

## Case presentation

A one-year-old male patient with no significant past medical or surgical history presented to the emergency department with an acute 24- to 48-hour history characterized by an inability to tolerate oral intake, recurrent vomiting, progressive abdominal distension, and severe abdominal pain with irritability and signs of peritoneal irritation.

Laboratory studies did not reveal significant leukocytosis, showing a white blood cell count of 8.59 × 10³/μL with 72.7% neutrophils. C-reactive protein was elevated at 71 mg/L, while arterial blood gas revealed a pH of 7.40 and a lactate level of 0.9 mmol/L.

The abdominal computed tomography (CT) (Figure 1) scan performed at our institution revealed a bowel obstruction with a transition zone in the terminal ileum. Given the clinical deterioration and suspicion of an acute obstructive abdomen, urgent surgical management was undertaken via a supraumbilical transverse laparotomy.

**Figure 1:**
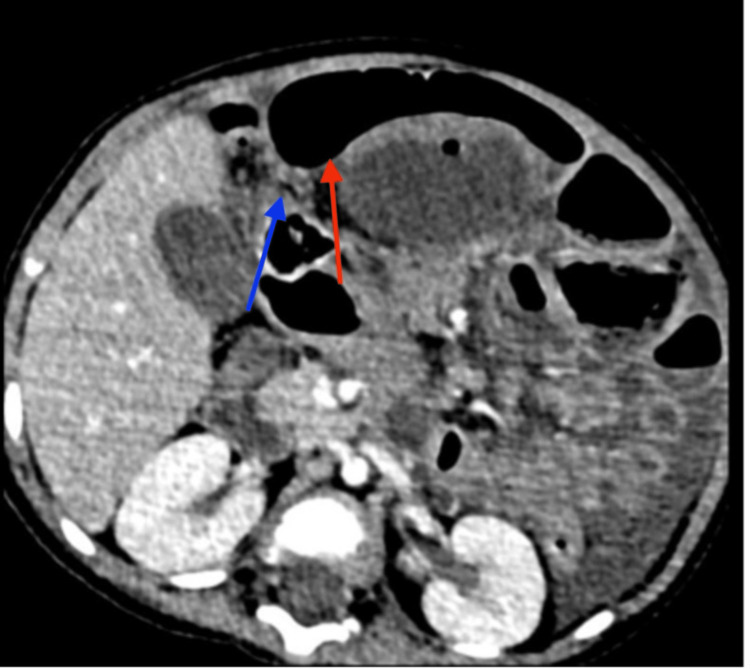
Abdominal computed tomography, note the dilation of the bowel loops. The abdominal computed tomography scan performed at our institution revealed a bowel obstruction (red arrow) with a transition zone (blue arrow) in the terminal ileum.

Intraoperative findings

Even though the first impression due to the CT scan was a volvulus, once we performed the laparotomy, the malrotation was evidenced.

An approximately 100-cm segment of ischemic terminal ileum (Figures 2, 3) was identified secondary to compression by Ladd's bands (Figure 2), located 20 cm proximal to the ileocecal valve.

**Figure 2:**
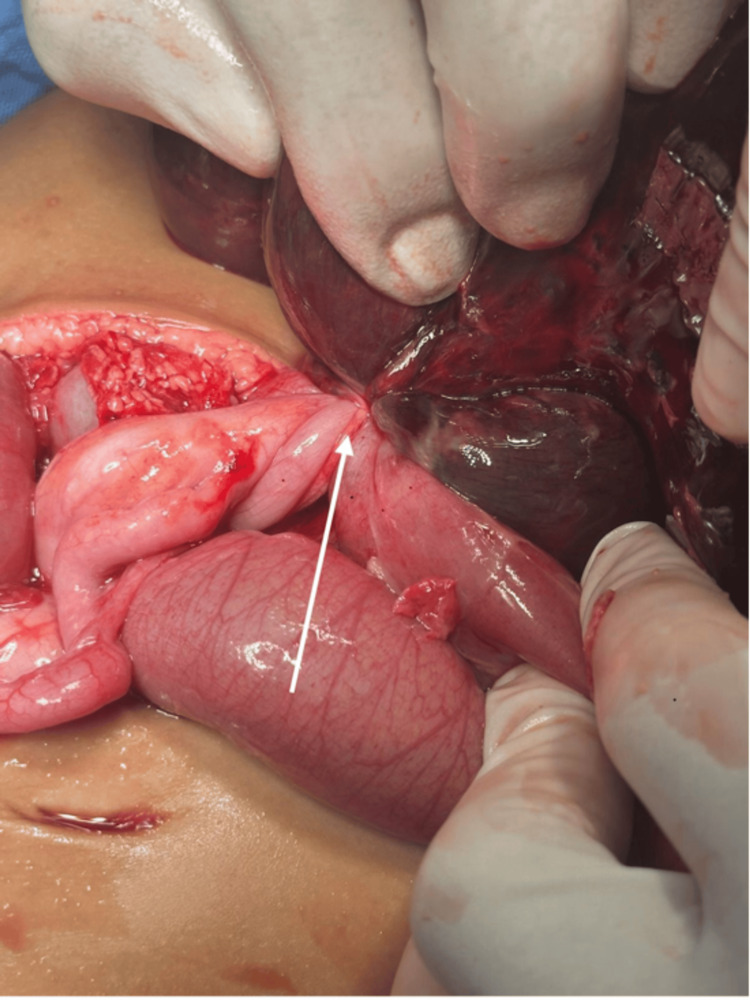
Intraoperative photograph showing Ladd's band (indicated by the arrow) compressing the small bowel.

**Figure 3:**
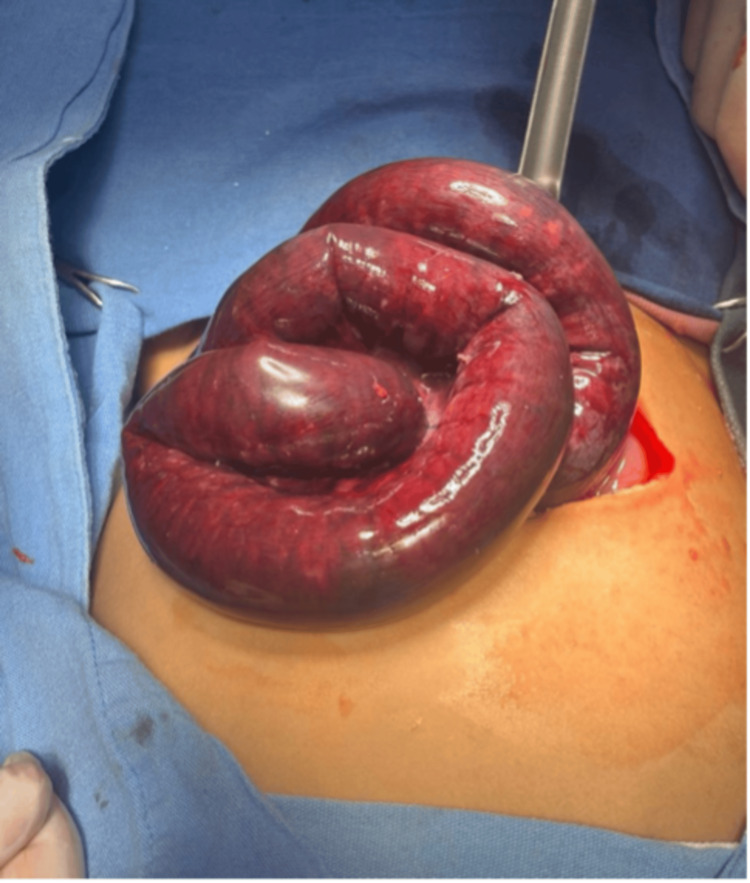
Intraoperative photograph of the ischemic bowel exhibiting violaceous discoloration and evident venous congestion, corresponding to the segment affected by the obstruction.

Following the principles of the Ladd's procedure, the bowel was repositioned in a non-rotated configuration, with the small intestine placed on the right side of the abdominal cavity and the colon positioned on the left side. Because of the extent of bowel ischemia and concerns regarding intestinal viability, intestinal continuity was not restored during the initial operation. Instead, an end ileostomy and a distal mucous fistula were created and matured in the right lower quadrant.

During the postoperative period, the patient remained NPO (nil per os) for the first 48 hours under strict fluid and electrolyte management. Subsequently, dietary progression to liquids was initiated, with good tolerance and adequate functioning of the ileostomy. The patient was discharged with outpatient follow-up for evaluation of bowel continuity restoration in no less than six months.

## Discussion

Intestinal malrotation represents an uncommon cause of bowel obstruction in pediatrics outside the neonatal period, although it carries a high risk of vascular compromise [5,6]. Normal intestinal rotation during embryogenesis is described as occurring between weeks 4 and 10 of gestation. This pathology occurs in approximately 1 out of every 200 to 500 live births; only about 0.2% of cases are diagnosed in adulthood [7,8]. Symptomatology can vary from intermittent presentations to complete obstruction, causing increased intraluminal pressure with subsequent progression to ischemia, as seen in this case.

Because the consequences of malrotation associated with a midgut volvulus may be catastrophic, an understanding of the anatomy, diagnostic criteria, and appropriate therapy for this putative emergency illness is imperative [9].

A noteworthy aspect is that, despite the extensive ischemia, the patient presented with normal lactate levels and an absence of severe metabolic acidosis. This highlights that systemic markers of hypoperfusion do not always reliably reflect localized intestinal compromise -- a critical point to emphasize to avoid delayed diagnosis and subsequent complications.

At laparotomy, malrotation and ischemic terminal ileum were identified approximately 20 cm proximal to the ileocecal valve. Fibrous Ladd's bands extending from the abnormally positioned cecum to the retroperitoneum were identified and divided, releasing the intestinal obstruction. The mesenteric base was widened by mobilizing the duodenum and right colon to reduce the risk of future midgut volvulus. Due to the presence of irreversible ischemia, approximately 100 cm of terminal ileum was resected. An appendectomy was subsequently performed.

The surgical treatment of choice is the Ladd's procedure, which includes lysis of the bands, broadening of the mesenteric base, and an appendectomy (performed because the appendix ends up in an abnormal anatomical position). In cases with extensive necrosis, such as the one reported here, bowel resection and a temporary ostomy are required. The long-term sequelae of Ladd’s procedure and its complications are not well defined [10].

## Conclusions

Although Ladd's bands associated with intestinal malrotation represent a potentially catastrophic cause of bowel obstruction and intestinal ischemia in the pediatric population, early clinical suspicion, the timely use of diagnostic imaging, and immediate surgical intervention are decisive factors in improving prognosis, preventing complications, and minimizing the risk of extensive bowel resection.
